# HIV prevalence, testing and treatment among men who have sex with men through engagement in virtual sexual networks in Kenya: a cross‐sectional bio‐behavioural study

**DOI:** 10.1002/jia2.25516

**Published:** 2020-06-26

**Authors:** Parinita Bhattacharjee, Shajy Isac, Helgar Musyoki, Faran Emmanuel, Kennedy Olango, Samuel Kuria, Martin K Ongaro, Jeffrey Walimbwa, Janet Musimbi, Mary Mugambi, Shem Kaosa, Japheth Kioko, Margret Njraini, Memory Melon, Juddie Onyoni, Kigen Bartilol, Marissa Becker, Robert Lorway, Michael Pickles, Stephen Moses, James Blanchard, Sharmistha Mishra

**Affiliations:** ^1^ Centre for Global Public Health University of Manitoba Winnipeg Canada; ^2^ Technical Support Unit Partners for Health and Development in Africa Nairobi Kenya; ^3^ India Health Action Trust New Delhi India; ^4^ National AIDS and STI Control Programme Ministry of Health Nairobi Kenya; ^5^ Men Against AIDS Youth Group Kisumu Kenya; ^6^ Mamboleo Peer Empowerment Group Kiambu Kenya; ^7^ HIV and AIDS People’s Alliance of Kenya Mombasa Kenya; ^8^ G10 Research Advisory Committee Nairobi Kenya; ^9^ Imperial College London United Kingdom; ^10^ St. Michael’s Hospital Department of Medicine University of Toronto Toronto Canada; ^11^ Institute of Medical Sciences University of Toronto Toronto Canada; ^12^ Institute of Health Policy Management and Evaluation Dalla Lana School of Public Health University of Toronto Toronto Canada

**Keywords:** HIV prevalence, HIV testing, men who have sex with men, virtual networks, Kenya, HIV care continuum

## Abstract

**Introduction:**

In Kenya, men who have sex with men (MSM) are increasingly using virtual sites, including web‐based apps, to meet sex partners. We examined HIV testing, HIV prevalence, awareness of HIV‐positive status and linkage to antiretroviral therapy (ART), for HIV‐positive MSM who solely met partners via physical sites (PMSM), compared with those who did so in virtual sites (either solely via virtual sites (VMSM), or via both virtual and physical sites (DMSM)).

**Methods:**

We conducted a cross‐sectional bio‐behavioural survey of 1200 MSM, 15 years and above, in three counties in Kenya between May and July 2019, using random sampling of physical and virtual sites. We classified participants as PMSM, DMSM and VMSM, based on where they met sex partners, and compared the following between groups using chi‐square tests: (i) proportion tested; (ii) HIV prevalence and (iii) HIV care continuum among MSM living with HIV. We then performed multivariable logistic regression to measure independent associations between network engagement and HIV status.

**Results:**

177 (14.7%), 768 (64.0%) and 255 (21.2%), of participants were classified as PMSM, DMSM and VMSM respectively. 68.4%, 70.4% and 78.5% of PMSM, DMSM and VMSM, respectively, reported an HIV test in the previous six months. HIV prevalence was 8.5% (PMSM), 15.4% (DMSM) and 26.7% (VMSM), *p* < 0.001. Among those living with HIV, 46.7% (PMSM), 41.5% (DMSM) and 29.4% (VMSM) were diagnosed and aware of their status; and 40.0%, 35.6% and 26.5% were on antiretroviral treatment. After adjustment for other predictors, MSM engaged in virtual networks remained at a two to threefold higher risk of prevalent HIV: VMSM versus PMSM (adjusted odds ratio 3.88 (95% confidence interval (CI) 1.84 to 8.17) *p* < 0.001); DMSM versus PMSM (2.00 (95% CI 1.03 to 3.87), *p* = 0.040).

**Conclusions:**

Engagement in virtual networks is associated with elevated HIV risk, irrespective of individual‐level risk factors. Understanding the difference in characteristics among MSM‐seeking partners in different sites will help HIV programmes to develop subpopulation‐specific interventions.

## 1 INTRODUCTION

Globally, men who have sex with men (MSM) shoulder a high burden of HIV [1], with HIV acquisition risk 28 times higher than among men who only have sex with women [2]. In Kenya, bio‐behavioural surveillance studies among MSM in 2010 (the most recent national data available) documented an HIV prevalence of 18.9% [3], whereas the HIV prevalence among men in the general population in Kenya, in 2018, was 3.1% [4]. Thus, MSM comprise a priority population in Kenya’s national HIV response, despite the criminalization of same sex relationships [5].

There are more than 30 MSM‐focused HIV service providers (non‐governmental and community‐based organizations) in 33 of the 47 counties in Kenya [6]. They work in partnership with the National AIDS and STI Control Programme (NASCOP) and the National AIDS Control Council under the Ministry of Health. Services related to HIV care include HIV prevention, testing and linkage to HIV treatment, provided by MSM‐focused clinics or government clinics, as preferred by the client [7]. In 2012, geographical “hotspot” mapping and population size estimation of MSM in Kenya found that MSM primarily met male sex partners in physical spaces such as venues (bars, clubs), streets, homes and sex dens [8]. Accordingly, MSM‐focused HIV services were largely designed around outreach at these physical sites [7].

In Kenya, as elsewhere in sub‐Saharan Africa, and in other low and middle income settings, MSM are increasingly using virtual, web‐based apps and social network sites to meet male sex partners [9, 10, 11, 12, 13]. Studies conducted in Malawi, Namibia and Botswana in 2008 showed that in a pooled analysis, 44.7% of MSM had used the Internet to find male sexual partners in the last six months [14]. Similar studies conducted in 2011 in Eswatini, 2014 in Lesotho and 2015 in Nigeria, respectively, reported that 39%, 44% and 62% of MSM met new male sex partners online [10, 15]. In Kenya, Internet‐based mapping conducted by NASCOP in 2018 found that 25% of MSM sought male sexual partners solely in virtual sites, and thus would be missed in mapping and estimation that only included physical sites [16].

As indicated earlier, studies from sub‐Saharan Africa suggest that HIV prevalence is higher among MSM who seek partners in virtual sites than in physical sites, and that a large proportion are unaware of their HIV‐positive status [14, 15]. There are no data from Kenya comparing HIV risk or engagement in HIV services by MSM who met their partners in virtual as opposed to physical networks. There is also a concern that men who solely engage in virtual networks to meet sex partners may be less visible to existing MSM‐focused service providers, because services generally rely on outreach to physical sites [17]. At present in Kenya, MSM‐focused service providers do not systematically leverage online platforms to reach MSM at scale, although a few pilot projects have tested specific online platforms to reach MSM who use virtual sites [18].

Thus, to adapt MSM‐focused HIV prevention and care services in response to the changing landscape of how men meet other men for sex, it is important to understand whether and how MSM who meet other male sex partners solely in physical spaces (PMSM), may differ from those who meet partners in both physical and virtual spaces (dual network, DMSM), and MSM who solely meet sex partners in virtual spaces (VMSM), with respect to socio‐demographic characteristics, sexual behaviour and HIV prevention and care related practices. Our aims were to compare the following parameters between the three groups: (i) engagement in HIV services, including HIV testing; (ii) HIV prevalence and (iii) engagement in the HIV care continuum (awareness of HIV‐positive status and linkage to antiretroviral therapy (ART) for people living with HIV (PLHIV). We then sought to estimate the independent risk of HIV by network type, after accounting for individual‐level predictors of prevalent HIV infection among MSM in Kenya [19, 20, 21, 22].

## 2 METHODS

### 2.1 Study setting

This community‐based study was conducted in three counties in Kenya: Kisumu, Mombasa and Kiambu, representing the western, coastal and central regions of Kenya respectively.

### 2.2 Study design and participants

We used data from a cross‐sectional bio‐behavioural survey conducted among 1200 MSM recruited from virtual and physical sites in the three counties, from May to July 2019, as part of the baseline for an evaluation of HIV self‐testing implementation strategies among MSM [23]. Participants were included if they: (a) identified as male; (b) reported engaging in anal or oral sex with another male in the previous 12 months; and (c) were of 15 years of age or above. The study was co‐designed with community researchers and community‐based organizations in each of the three counties [23]. A multi‐stage cluster sampling approach involving physical and virtual sites was used to recruit 1200 participants. Programmatic mapping and enumeration was conducted in physical sites and in virtual sites where MSM met other male sex partners, to generate the sampling frame [16, 24]. A sample size of 1200 (400 in each county) was calculated to observe, with 80% power, a 10% to 15% absolute difference in the proportion of MSM living with HIV who are diagnosed and aware of their HIV status, between a baseline and a post‐intervention survey. After stratifying by county, we separately sampled sites to recruit 200 MSM from physical sites and 200 MSM from virtual sites in each county. Recruitment via physical sites involved random sampling of sites, within which two participants were randomly selected for recruitment. Each eligible participant who consented to participate then provided a list of all known contacts that identify as MSM, from which a random sample of one contact was selected for peer‐recruitment into the study. Recruitment via virtual site sampling was based on the estimated number of MSM who met sex partners using each virtual site within each county. Virtual sites were selected via random sampling, and peer researchers used each randomly selected virtual site to further randomly recruit the pre‐defined number of potential participants who were online when the peer researcher logged into the site. As with physical sites, each consented participant from the virtual sites provided a list of all known contacts that identify as MSM, from which a random sample of one contact was selected for peer‐recruitment into the study. The methodology is described in detail elsewhere [23].

### 2.3 Data collection

Data collection took place in private spaces (e.g. community‐based organizations, offices, drop in centres and clinics), at a time and location that was convenient to the participant. Individuals who met eligibility criteria were requested to visit a specified data collection site, where they were invited to provide informed, written consent, and they could choose to participate in all or some elements of the baseline bio‐behavioural survey. Trained researchers administered a face‐to‐face structured questionnaire (Appendix S1) in Kiswahili or English, as preferred by the respondent. All participants were offered HIV testing and counselling with a rapid two‐test algorithm as per Kenya national guidelines, with onsite reporting of results. If their HIV test was positive or inconclusive, participants were also offered accompanied referral to an MSM‐focused clinic, or to a government testing and treatment clinic. All participants were provided with condoms and lubricants, and given information on HIV self‐testing. Those who were seronegative were also offered referral for HIV pre‐exposure prophylaxis through local MSM‐focused clinics. Participants were also asked to provide a dried blood spot for HIV confirmatory serology, performed at the HIV National Laboratory in Nairobi, using the Bioelisa HIV test kit for screening and if positive, the Murex HIV1‐2‐O test for confirmation. Completed questionnaires were transferred to Nairobi and data were entered into a database developed using Census and Survey Processing System software (CSPro, US Census Bureau and ICF International). The data collection process is detailed further in the study protocol paper [22].

### 2.4 Measurement and data analysis

To define our three groups of interest, we used the following question (Appendix S1): “which are the different places/locations through which you have met other male sexual partners? (more than one option possible)” (question 17). If responses included “other,” researchers asked the respondent to specify, and places/locations were classified as virtual or physical sites during the analysis. Locations such as Internet/web app, Facebook, WhatsApp, mobile were categorized as virtual sites; and street, home, bus/taxi stand/lodge/markets/social gatherings were categorized as physical sites. Participants who reported using only physical sites as locations through which they met other male sexual partners were defined as physical site MSM (PMSM). Participants who reported using dual sites, that is both physical sites and virtual sites as locations through which they met male sexual partners, were defined as dual site MSM (DMSM). Participants who self‐reported only virtual sites as locations through which they met other male sex partners were defined as virtual site MSM (VMSM).

Socio‐demographic characteristics included current age, highest level of educational attainment and monthly income. Variables related to sexual behaviour included: preferred sexual position/role; age at first anal/oral sex with a man; duration in years since first anal/oral sex with a man; number of different male partners in the past one month; receipt of money or gifts in exchange for sex with a man and condom use at last sex with a male partner. The two measures of engagement with MSM‐focused HIV services were as follows: contact by a peer educator in the prior three months; and visit to an MSM‐focused clinic/drop‐in centre in the previous three months. HIV testing was measured via self‐reported “ever” tested for HIV, and self‐reported HIV test in the previous twelve, six and three months.

We compared available HIV “cascade/care continuum” indicators among those living with HIV: the proportion diagnosed and aware of their results at the time of the survey; the proportion registered in an HIV care and treatment programme; those ever on ART; those currently on ART; and among those currently on ART, the proportion who missed taking ART last month. Among participants living with HIV, those who reported their HIV status as HIV‐negative, or did not know, or did not want to disclose, were classified as undiagnosed and unaware. We used chi‐square tests for comparison of proportions, and the Kruskal–Wallis H non‐parametric test to compare medians.

To determine if engagement in a particular sex network was independently associated with prevalent HIV infection, we performed multivariate logistic regression using HIV infection (based on the dried blood spot serology result) as the outcome variable. Our main exposure variable of interest was sole engagement in physical sites, dual site engagement or sole engagement in virtual sites. We adjusted for county and for potential individual‐level confounders for prevalent HIV infection based on prior literature [19, 20, 21, 22]: socio‐demographic characteristics (age, educational attainment); and sexual behaviour and partnership characteristics. The unadjusted and adjusted analyses excluded 11 participants who responded “other” or “no answer” to the question related to preference with respect to sexual position/role. We have presented the crude and adjusted odds ratios (ORs) of prevalent HIV infection among VMSM and for DMSM compared to PMSM; and the corresponding 95% confidence intervals (CIs). Data were analysed using SPSS 25 (IBM SPSS Statistics for Windows, Version 25.0. Armonk, NY: IBM Corp.).

### 2.5 Ethics approval

The study received ethics approval from the institutional review boards of the Kenyatta National Hospital – University of Nairobi, Kenya (P557/08/2018) and the University of Manitoba – Health Research Ethics Board, Winnipeg, Canada (HS22205).

## 3 RESULTS

### 3.1 Comparison of socio‐demographic and sexual behaviour

The non‐response rate from sampling of physical sites was 13%, but was not documented from the sampling of virtual sites (Table 1). Overall, 177/1200 (14.7%) of participants reporting solely using physical sites (PMSM), 768/1200 (64.0%) reported using dual sites (DMSM) and 255/1200 (21.2%) reported solely using virtual sites (VMSM). The socio‐demographic and sexual behaviour characteristics by network engagement are depicted in Table 1.

**Table 1:** Comparison of socio‐demographic and sexual behaviour among MSM by network type in Kenya, May to July 2019

	Total	PMSM^a^	DMSM^a^	VMSM^a^	*p* value
	N = 1200 (%)	N = 177 (%)	N = 768 (%)	N = 255 (%)	
Age (years)					
Median (IQR)	23 (21.0 to 27.0)	23.0 (21.0 to 27.0)	24.0 (21.0 to 27.0)	23.0 (21.0 to 27.0)	0.022
<25 years	61.1%	59.3%	60.2%	65.1%	0.327
25 + years	38.9%	40.7%	39.8%	34.9%	
Highest level of educational attainment					
Up to primary	19.8%	27.1%	21.9%	8.6%	0.000
Secondary	49.4%	50.3%	49.3%	49.0%	
Post – secondary^b^	30.8%	22.6%	28.8%	42.4%	
Monthly income					
Median (IQR)	8000 (0 to 15000)	8000 (4000 to 15000)	9000 (2000 to 15000)	0.0 (0 to 16000)	0.004
No income	29.3%	19.8%	24.0%	52.2%	0.000
<10000 Shilling/<100 USD	24.3%	36.2%	27.0%	7.8%	
10000 + Shilling/100 + USD	46.4%	44.1%	49.1%	40.0%	
Preference with respect to sexual position/role					
Predominantly receptive (bottom)	21.8%	18.1%	20.1%	29.4%	0.002
Predominantly insertive (top)	45.8%	56.5%	45.2%	40.0%	
Both receptive and penetrative	31.6%	24.9%	33.6%	30.2%	
Others/non‐answer	0.9%	0.6%	1.2%	0.4%	
Age at first anal/oral sex with a man (years)					
Median (IQR)	18.0 (16.0 to 20.0)	18.0 (16.0 to 22.0)	18.0 (16.0 to 20.0)	18.0 (17.0 to 20.0)	0.025
<15 years	10.3%	7.3%	12.4%	6.3%	0.003
15 to 17 years	34.5%	33.3%	36.1%	30.6%	
18 + years	55.2%	59.3%	51.6%	63.1%	
Duration since first had anal/oral sex with another man (years)					
Median (IQR)	5.0 (3.0 to 8.0)	4.0 (2.0 to 7.0)	5.0 (3.0 to 9.0)	4.0 (2.0 to 7.0)	0.020
<5 years	46.0%	53.7%	42.0%	52.8%	0.001
5 to 9 years	34.2%	30.5%	35.1%	34.3%	
10 + years	19.8%	15.8%	22.9%	13.0%	
Number of different male sexual partners in the past one month					
Median (IQR)	2.0 (1.0 to 3.0)	1.0 (1.0 to 3.0)	2.0 (1.0 to 3.0)	2.0 (1.0 to 3.0)	0.832
<2	41.1%	58.9%	37.6%	39.2%	0.000
2+	58.9%	41.1%	62.4%	60.8%	
Received money/gift in exchange of sex with man	59.9%	64.4%	66.0%	38.4%	0.000
Condom use with last male sexual partner	71.8%	67.0%	73.1%	71.1%	0.260

IQR, inter‐quartile range; MSM, men who have sex with men.

^a^ PMSM (physical‐space only network); DMSM (dual network); VMSM (virtual‐space only network). Participants who self‐reported only virtual sites (Internet/web app, Facebook, WhatsApp, mobile) as locations through which they met other male sex partners were defined as MSM who met sex partners solely using virtual sites (VMSM). Participants who reported using only physical sites (street, home, bus/taxi stand/lodge/markets/social gatherings) as locations through which they met other male sexual partners were defined as MSM who met sex partners solely using physical sites (PVSM). Participants who reported using both physical sites (street, home, bus/taxi stand/lodge/markets/social gatherings) and virtual sites (Internet/web app, Facebook, WhatsApp, mobile) as locations through which they met male sexual partners were defined as MSM who met sex partners using dual sites (DMSM);

^b^ post‐secondary (tertiary/college/university).

Although the age distribution across groups was similar, VMSM were more likely to report higher educational attainment (42.4% reported post‐secondary as their highest level of education, compared with 22.6% of PMSM and 28.8% of DMSM, *p* < 0.001); and to report zero monthly income (52.2% of VMSM vs. 19.8% of PMSM and vs. 24.0% of DMSM, *p* < 0.001). There was notable variability in preferred sexual position or role, as a higher (29.4%) of VMSM preferred a predominant receptive anal sex role, compared with 18.1% of PMSM and 20.1% of DMSM, *p* = 0.002. The median age at first anal/oral sex with a man was similar across groups, but the age distribution was different, with a slightly higher proportion (63.1%) of VMSM reporting first sex after age 18 years, as compared with PMSM (59.3%) and DMSM (51.6%), *p* = 0.003. Accordingly, and given a similar distribution in current age, VMSM reported a shorter duration since first sex with another man. The median number of male sex partners in the past month was similar across groups, but a higher proportion of VMSM (60.8%) and DMSM (62.4%) reported more than two sex partners, as compared with PMSM (41.1%, *p* < 0.001). Fewer (38.4%) of VMSM reported receiving money/gifts in exchange for sex with men, as compared with 64.4% of PMSM and 66.0% of DMSM (*p* < 0.001). The proportion of men who reported condom use with last male sex partner was similar across groups.

### 3.2 Comparison of engagement in MSM‐focused HIV services and HIV testing

Half of VMSM (50.2%) and DMSM (51.3%) had been contacted by a peer educator or outreach worker from an MSM‐focused HIV service in the previous three months, as compared with only 36.0% of PMSM (*p* < 0.001) (Table 2). Similarly, half of VMSM (48.6%) and DMSM (49.0%) had visited an MSM‐focused HIV clinic or drop‐in centre in the previous three months, compared with only 31.1% of PMSM (*p* < 0.001). Nearly all respondents reported a prior HIV test (97.0%), and HIV testing in the past 12 months was also high (85.1%), and similar across groups. However, the likelihood of recent testing was highest in VMSM, followed by DMSM, and lowest in PMSM: 78.5%, 70.4% and 68.4% were tested in the previous six months, *p* = 0.03; and 68.7%, 58.2% and 57.3 were tested in the previous three months, *p* = 0.01. With respect to their most recent HIV test, 20.0%, 29.63% and 40.2% of PMSM, DMSM and VMSM had been tested in an MSM‐focused clinic (*p* < 0.001, Table S1).

**Table 2:** Engagement in MSM‐focused HIV services and HIV testing among MSM by network type in Kenya, May to July 2019

	Total	PMSM^a^	DMSM^a^	VMSM^a^	*p* value
Contacts with MSM‐focused HIV programme					
Contacted by a peer educator or outreach worker in the past three months (N = 1194)	48.8%	36.0%	51.3%	50.2%	0.001
Visited an MSM‐focused HIV programme clinic or drop‐in centre in the past three months (N = 1199)	46.3%	31.1%	49.0%	48.6%	0.001
HIV testing					
Ever tested for HIV (N = 1200)	97.0%	95.5%	97.4%	96.9%	0.400
Tested for HIV in the past 12 months (N = 1184)	85.1%	82.2%^b^	84.5%^c^	89.1%^d^	0.104
Tested for HIV in the past six months (N = 1170)	71.8%	68.4%^b^	70.4%^c^	78.5%^d^	0.029
Tested for HIV in the past three months (N = 1153)	60.3%	57.3%^b^	58.2%^c^	68.7%^d^	0.010

MSM, men who have sex with men.

^a^ PMSM (physical‐space only network); DMSM (dual network); VMSM (virtual‐space only network). Participants who self‐reported only virtual sites (Internet/web app, Facebook, WhatsApp, mobile) as locations through which they met other male sex partners were defined as MSM who met sex partners solely using virtual sites (VMSM). Participants who reported using only physical sites (street, home, bus/taxi stand/lodge/markets/social gatherings) as locations through which they met other male sexual partners were defined as MSM who met sex partners solely using physical sites (PVSM). Participants who reported using both physical sites (street, home, bus/taxi stand/lodge/markets/social gatherings) and virtual sites (Internet/web app, Facebook, WhatsApp, mobile) as locations through which they met male sexual partners were defined as MSM who met sex partners using dual sites (DMSM);

^b^ among PMSM, the denominator was N = 174, N = 171 and N = 171, for individuals tested in the past twelve, six and three months respectively. Individuals reported that in their most recent HIV test at >12, >6 months ago as positives were removed from the numerator and denominator of the analysis of >6 and >3 months respectively;

^c^ among DMSM, the denominator was N = 762, N = 753 and N = 739, for individuals tested in the past twelve, six and three months respectively. Individuals reported that in their most recent HIV test at >12, >6 months ago as positives were removed from the numerator and denominator of the analysis of >6 and >3 months respectively;

^d^ among VMSM, the denominator was N = 248, N = 246 and N = 243, for individuals tested in the past twelve, six and three months respectively. Individuals reported that in their most recent HIV test at >12, >6 months ago as positives were removed from the numerator and denominator of the analysis of >6 and >3 months respectively.

### 3.3 Comparison of the HIV cascade/care continuum

HIV prevalence was highest in VMSM (26.7%, 95% CI: 21.2 to 32.1), followed by DMSM (15.4% 95%, CI: 12.8 to 17.9) and lowest in PMSM (8.5%, 95% CI: 4.3 to 12.6; *p* < 0.001) (Table 3). Among those living with HIV, 46.7% (PMSM), 41.5% (DMSM) and 29.4% (VMSM) were diagnosed and aware of their HIV status (*p* = 0.220). Of the 201 participants living with HIV, 32.8% were registered at an HIV treatment and care centre, all of whom had “ever initiated on ART” and 32.3% were currently on ART (40.0% in PMSM, 35.6% in DMSM and 25.0% of VMSM, *p* = 0.270). However, of those on ART, 50.0% of PMSM, 60.5% of DMSM and 88.2% of VMSM never missed taking their ARV in the past one month, *p* = 0.08).

**Table 3:** HIV prevalence and HIV cascade among MSM living with HIV by network type in Kenya, May to July 2019

	Total (N = 1200)	PMSM^a^ (N = 177)	DMSM^a^ (N = 768)	VMSM^a^ (N = 255)	*p* value
	% [95% CI]	% [95% CI]	% [95% CI]	% [95% CI]	
HIV prevalence^b^	16.8 [14.6 to 18.9]	8.5 [4.3 to 12.6]	15.4 [12.8 to 19.9]	26.7 [21.2 to 32.1]	0.000

	Total MSM living with HIV^b^ (N = 201)	PMSM^a^ (N = 15)	DMSM^a^ (N = 118)	VMSM^a^ (N = 68)	*p* value
MSM living with HIV aware of HIV‐positive status^c^	37.8%	46.7%	41.5%	29.4%	0.220
Registered in HIV treatment and care centre	32.8%	40.0%	35.6%	26.5%	0.367
Ever on ART	32.8%	40.0%	35.6%	26.5%	0.367
Currently on ART	32.3%	40.0%	35.6%	25.0%	0.266
Never missed taking ARV in the past one month (of those currently on ART) (N = 66)	66.7%	50.0%	60.5%	88.2%	0.080

ART, antiretroviral treatment; MSM, men who have sex with men

^a^ PMSM (physical‐space only network); DMSM (dual network); VMSM (virtual‐space only network). Participants who self‐reported only virtual sites (Internet/web app, Facebook, WhatsApp, mobile) as locations through which they met other male sex partners were defined as MSM who met sex partners solely using virtual sites (VMSM). Participants who reported using only physical sites (street, home, bus/taxi stand/lodge/markets/social gatherings) as locations through which they met other male sexual partners were defined as MSM who met sex partners solely using physical sites (PVSM). Participants who reported using both physical sites (street, home, bus/taxi stand/lodge/markets/social gatherings) and virtual sites (Internet/web app, Facebook, WhatsApp, mobile) as locations through which they met male sexual partners were defined as MSM who met sex partners using dual sites (DMSM);

^b^ HIV serology based on the dried blood test;

^c^ aware of HIV status based on self‐reported positive status as per the last test HIV prior to the study and the test was HIV positive. Individuals who had “never tested for HIV” were categorized as not aware. All respondents who had “ever tested for HIV” and disclosed their last HIV test result to the interviewer were considered as aware of HIV status.

### 3.4 Association between network engagement and prevalent HIV infection

Compared with PMSM, DMSM were at a twofold (unadjusted OR 1.96, 95% CI: 1.12 to 3.45) higher risk of a prevalent HIV infection; and VMSM were at a fourfold higher risk (unadjusted OR 3.93, 95% CI: 2.16 to 7.14) (Table 4). The association between network type and HIV persisted in direction and magnitude after adjusting for county and for potential individual‐level confounders: VMSM versus PMSM (adjusted OR 3.88, 95% CI: 1.84 to 8.17) and DMSM versus PMSM (adjusted OR 2.00, 95% CI: 1.03 to 3.87).

**Table 4:** Independent association between network type and HIV after adjusting for county and individual – level risk factors for HIV among MSM in Kenya, May to July 2019

	Prevalence of HIV infection (N = 197/1200)^a^			
	Unadjusted		Adjusted	
	Odds ratio [95% CI]	*p* value	Odds ratio [95% CI]	*p* value
Network^b^				
PMSM	1	Ref	1	Ref
VMSM	3.93 [2.16 to 7.14]	0.000	3.26 [1.60 to 6.67]	0.001
DMSM	1.96 [1.12 to 3.45]	0.019	1.79 [0.95 to 3.36]	0.072
County				
Kisumu	1	Ref	1	Ref
Mombasa	2.57 [1.66 to 3.97]	0.000	1.46 [0.88 to 2.43]	0.141
Kiambu	3.37 [2.20 to 5.15]	0.000	2.11 [1.26 to 3.51]	0.004
Age (years)^c^	1.10 [1.07 to 1.13]	0.000	1.13 [1.09 to 1.17]	0.000
Highest level of Education attainment				
Up to primary	1	Ref	1	Ref
Secondary	0.63 [0.42 to 0.93]	0.020	0.85 [0.53 to 1.37]	0.509
Post – secondary^d^	0.96 [0.64 to 1.44]	0.843	1.24 [0.74 to 2.06]	0.412
Monthly income^c^	1.00 [1.00 to 1.00]	0.085	1.00 [1.00 to 1.00]	0.905
Preference with respect to sexual position				
Insertive (top)	1	Ref	1	Ref
Receptive (bottom)	2.73 [1.82 to 4.11]	0.000	2.59 [1.67 to 4.02]	0.000
Both	2.85 [1.96 to 4.13]	0.000	2.26 [1.51 to 3.37]	0.000
Age at first anal/oral sex with a man (years)^c^	0.98 [0.94 to 1.02]	0.351	0.94 [0.90 to 0.98]	0.003
Number of male sexual partners in the past one month^c^	1.08 [1.03 to 1.14]	0.002	1.04 [0.98 to 1.10]	0.192
Receive money/gift in exchange of sex with another man^e^				
No	1	Ref	1	Ref
Yes	0.97 [0.71 to 1.32]	0.843	1.03 [0.70 to 1.51]	0.889

CI, confidence interval; MSM, men who have sex with men.

^a^ HIV serology based on the dried blood test. Analyses excludes N = 11 respondents who responded as “other” or “no answer” to question related to preference with respect to sexual position;

^b^ PMSM (physical‐space only network); DMSM (dual network); VMSM (virtual‐space only network). Participants who self‐reported only virtual sites (Internet/web app, Facebook, WhatsApp, mobile) as locations through which they met other male sex partners were defined as MSM who met sex partners solely using virtual sites (VMSM). Participants who reported using only physical sites (street, home, bus/taxi stand/lodge/markets/social gatherings) as locations through which they met other male sexual partners were defined as MSM who met sex partners solely using physical sites (PVSM). Participants who reported using both physical sites (street, home, bus/taxi stand/lodge/markets/social gatherings) and virtual sites (Internet/web app, Facebook, WhatsApp, mobile) as locations through which they met male sexual partners were defined as MSM who met sex partners using dual sites (DMSM);

^c^ included as continuous variable;

^d^ post‐secondary (tertiary/college/university);

^e^ asked as “do you receive money/gifts in exchange for sex with another man?”

## 4 DISCUSSION

Our cross‐sectional study identified a large subset of MSM in Kenya who use both virtual and physical sites to meet sex partners; and smaller, but important subsets who only use virtual sites or only physical sites. A surprising finding was that recent engagement with MSM‐focused HIV services and recent HIV testing was more commonly reported by men engaged in virtual networks (either solely or as part of a dual network). However, engagement in virtual networks was associated with a two to fourfold higher risk of prevalent HIV infection compared with sole engagement in physical sites, which could not be explained by geography nor by individual‐level risk factors examined in the study.

The use of virtual sites to meet partners was more commonly reported in our study (85% of MSM overall, including those who exclusively used virtual sites and those who used physical sites as well) than in other studies across sub‐Saharan Africa [10, 14, 15]. This may reflect differences in socio‐political and Internet availability, but also temporal differences, as suggested from other high income settings, where the use of virtual spaces has increased over time [25].

There were notable differences between the three networks. Chief among them was higher educational attainment, yet higher levels of zero income among VMSM. We hypothesize that this may be because VMSM include a large proportion of (unemployed) college or other students; however, we did not ask about current educational enrolment in the survey. The educational profile of VMSM in our study is similar to the educational profile of MSM engaged in virtual networks in Lesotho, Eswatini and Nigeria [10, 15]. The sexual behaviour of VMSM and of DMSM included factors known to be associated with both higher and lower HIV risk. Our findings show that MSM who seek sexual partners in virtual sites (either solely or along with physical sites) preferred receptive anal sex role and had more sexual partners. Studies from Asia and United States have shown that MSM who seek sexual partners on the Internet had more homosexual partners, similar to our study [12].

Our finding of an elevated risk of HIV in MSM who meet sex partners online is similar to findings from China [12], Malawi [14] and Nigeria [15]. Importantly, the individual‐level risk factors that we explored could not explain the difference between the two groups. One possible explanation for this could be that there are unexplained confounders such as the experience of violence or condomless anal sex, which can lead to increased individual‐level risk of HIV acquisition [18, 26]. Another reason could be that the structure of the sexual network itself increases the likelihood of acquiring HIV, even if all other individual‐level or partner‐level factors remain the same [27]. If a subset of MSM meet sex partners via virtual sites, then the likelihood that their sex partner is living with HIV is already two to three times that of MSM in other networks. Sexual network characteristics such as network structure, density, homophily and a person’s centrality within a sexual network play important roles in understanding HIV transmission among MSM [27, 28, 29].

The overall high proportion of undiagnosed HIV is consistent with findings from several countries in sub‐Saharan Africa [14], and is cause for concern when the proportion recently tested is moderately high. The reasons for low awareness could include acquisition of HIV after the last test or having tested during the window period and received a negative test result [30]. Some participants living with HIV may have been aware of their HIV diagnosis but did not wish to disclose in a face‐to‐face interview due to fear of stigma or discrimination [31] or social desirability bias. If VMSM and DMSM experience a higher incidence of HIV compared to PMSM (as the prevalence data would suggest), then HIV testing frequency among MSM in virtual networks should be further enhanced. Stanford et al. found that increased frequency of HIV testing was related to awareness of the HIV diagnosis [32]. Our findings also suggest that once MSM living with HIV are diagnosed, then initiation into ART, and adherence to ART programme, is good. Of note, VMSM were more likely to adhere to their ARVs, a finding that warrants further exploration [33]. Our findings also suggest that with new strong evidence around undetectable equals untransmittable [34], reducing the undiagnosed fraction among men engaged in virtual networks, along with their initiation and retention on ART, could be an important strategy for reducing onward transmission of infection.

These findings have several implications for HIV prevention and care programming for MSM. First, moving from individual‐level risk factors to considering networks – and thus, types of networks – could better identify subsets of MSM who remain most vulnerable to HIV acquisition and transmission. Network types, therefore, could be prioritized for HIV prevention and treatment, and MSM‐focused services could harness virtual sites to reach MSM [35, 36, 37, 38, 39, 40, 41, 42]. A systematic review of HIV intervention delivery among MSM in sub‐Saharan Africa identified pilot projects to reach MSM through the Internet in Ghana and the Democratic Republic of Congo [43]. Sexual networks are dynamic, and our findings signal the importance of leveraging how MSM connect with each other and the information platforms they use, to deliver rights‐based and effective HIV prevention and treatment interventions.

To date, few studies from low and middle income countries have compared MSM across such networks to characterize their profiles and their HIV risks and HIV prevalence [12, 44, 45]; to our knowledge, our study is one of the first from East Africa to do so. However, our study has important limitations. First, the data were collected via face‐to‐face interviews and thus are subject to social desirability bias, with the possibility of underreporting of higher risk practices or HIV status [46]. Second, our definitions of VMSM, PMSM and DMSM did not include a time‐frame, and so we could not distinguish men who used virtual sites for a short period of time from those who used them for several years. Third, we combined data from three counties, and although we adjusted for county when exploring the association between network engagement and HIV, further work is needed to characterize the sexual networks within each county. Fourth, the sampling method included multi‐stage sampling of physical sites and of virtual sites, which means that there could be some within‐site homogeneity in estimates because we recruited two seeds and two peers, we could not appropriately account for within‐cluster homogeneity in our analysis and thus may have overestimated differences between groups. The 2018 mapping and enumeration of MSM in Kenya signalled the importance of recruiting MSM from virtual sites (as 25% of MSM sought male sexual partners solely in the virtual sites), and thus it was critical to include virtual‐site sampling. However, there are no established approaches to doing so, and our approach (like those of others) might lead to selection bias. For example, participants from the virtual sites who are online at the same time might belong to the same subnetwork, and may therefore have similar characteristics. Our approach to virtual‐site sampling made it challenging to document a non‐response rate, which further limits our ability to judge selection bias. Fifth, we did not include sexual identity in our list of variables because we erroneously merged the categorical responses in the study tool. Sixth, we did not ask questions about current or recent schooling level, which may be important in understanding participants in virtual networks. Finally, we did not measure exposure to ART via biological sampling, and thus are limited to self‐reported ART status.

Further research is needed to understand the reasons for the high proportion of MSM who indicated that they were undiagnosed, even after moderately frequent testing. This could help us to understand the optimal frequency of HIV testing. Our findings also call for investigation into the structure and characteristics of virtual and dual networks, and how structures of networks might help to explain observed HIV prevalences. Studies in Kenya have shown that MSM also have female partners and engage in sex work [47]. Further research is needed to understand heterosexual sexual relationships among MSM engaged in virtual sites and their sex work‐related partnerships. Finally, we need implementation and programme science to assess how, when and under what contexts innovative network‐based interventions could be effectively delivered within virtual sites for MSM in Kenya, and across sub‐Saharan Africa.

## 5 CONCLUSIONS

Virtual spaces have become common ways to meet sex partners among MSM in Kenya, and are associated with a two to threefold greater risk of HIV compared to those using only physical sites. Hence, tailoring HIV‐related prevention, testing and treatment programmes to MSM using virtual sites should be an important focus for HIV prevention and care programmes.

Programmes need to better understand the heterogeneity in the MSM population and develop different service delivery models to enable: (a) effective reach of prevention interventions such as PrEP and condoms, (b) frequent testing and early diagnosis and (c) entry and retention in care and treatment.

## Competing interests

The authors declare that they have no competing interests.

## Authors’ contributions

PB, SI, FE, SMi and HM conceptualized the paper. PB, SI and SMi designed the plan of analysis. PB, SI and SMi wrote the first draft of the paper with edits from SI, RL, MB, MP, JB and SM. All authors contributed to questionnaire design, and interpretation of data and results; and all reviewed the manuscript and provided edits and suggestions. JB and PB conceptualized the larger study method. HM and PB generated the data, and JM, SK, JK, MM, MN and JO managed the data collection process. KO, SM, MOK, MM and JW supported the design of the study and on site data collection process. SI led questionnaire development and sampling design, and conducted the data analyses with input from PB and SMi. SM did the final edit of the manuscript. All authors have read and approved the final manuscript.

## Abbreviations

CBO, community‐based organization; CI, confidence interval; DMSM dual site MSM; MSM, men who have sex with men; NASCOP, National AIDS and STI Control Programme; OR, odds ratio; PMSM, physical site MSM; VMSM, virtual site MSM.

## Supporting information


**Appendix S1.** Survey questionnaire for baseline and end line survey.

**Table S1.** Consumption of drugs (oral or injecting) and place of most recent test among MSM who meet sex partners solely via virtual sites versus solely via physical sites versus via both physical and virtual sites in Kenya, May to July 2019

## References

[1] Beyrer C , Baral SD , Collins C , Richardson ET , Sullivan PS , Sanchez J , et al. The global response to HIV in men who have sex with men. Lancet. 2016;388(10040):198–206. 27411880 10.1016/S0140-6736(16)30781-4

[2] Joint United Nations . Programme on HIV/AIDS. Miles to go. Closing gaps, breaking barriers, righting injustices. Global AIDS Update. Geneva: UNAIDS 2018 [cited 2019 Aug 20]. Available from: https://www.unaids.org/sites/default/files/media_asset/miles‐to‐go_en.pdf

[3] National AIDS and STI Control Programme (NASCOP) . 2010‐2011 Integrated biological and behavioural surveillance survey among key populations in Nairobi and Kisumu, Kenya. Nairobi: Government of Kenya, Ministry of Public Health and Sanitation; 2014 [cited 2019 Aug 20]. Available from: https://globalhealthsciences.ucsf.edu/sites/globalhealthsciences.ucsf.edu/files/pub/final_report_keypops_ibbs_nov_24_2014_print.pdf

[4] National AIDS and STI Control Programme (NASCOP) . Preliminary KENPHIA 2018 Report. Nairobi: NASCOP; 2019 [cited 2020 Mar 25]. Available from: http://www.health.go.ke/wp‐content/uploads/2020/02/KENPHIA‐2018‐PREL‐REP‐2020‐HR3‐final.pdf

[5] National AIDS Control Council (NACC) . Kenya AIDS Strategic Framework 2013/4‐2018/19. Nairobi: Government of Kenya; 2014 [cited 2019 Aug 20]. Available from: http://nacc.or.ke/wp‐content/uploads/2015/09/KASF_Final.pdf

[6] National AIDS and STI Control Programme (NASCOP) . Quarterly Programme Reports, March 2019. Nairobi: Government of Kenya, 2019.

[7] National AIDS & STI Control Programme (NASCOP) . National guidelines for HIV/STI programming with key populations. Nairobi: Government of Kenya, 2014 [cited 2019 Aug 27]. Available from: http://www.icop.or.ke/wpcontent/uploads/2016/10/KP‐National‐Guidelines‐2014‐NASCOP.pdf

[8] National AIDS Control Council (NACC) and National AIDS and STI Control Programme (NASCOP) . Geographic mapping of most at risk populations for HIV in Kenya. Nairobi: Ministry of Health; Government of Kenya; 2012 [cited 2019 Sep 12] Available from: https://www.icop.or.ke/wp‐content/uploads/2016/09/Kenya‐mapping‐report‐NASCOP‐and‐UoM‐2013.pdf

[9] Wgenaar BH , Sullivan PS , Stephenson R . HIV knowledge and associated factors among internet using men who have sex with men (MSM) in South Africa and the United States. PLoS ONE. 2012;7:e32915. 22427908 10.1371/journal.pone.0032915 PMC3299717

[10] Stahlman S , Grosso A , Ketende S , Mothopeng T , Taruberekera N , Nkonyana J , et al. Characteristics of men who have sex with men in southern Africa who seek sex online: a cross‐sectional study. J Med Int Res. 2015;25(17):e129. 10.2196/jmir.4230 PMC4468572 26006788

[11] Bourne A , Tenza S , Liku J , Moodley K , Tukai A , Smith A , et al. The role of social media in sexual connectivity among men who have sex with men (MSM) in Kenya and South Africa: potential for innovative online sexual health promotion interventions. Poster presentation. 22nd International AIDS Conference (AIDS 2018), Amsterdam, Netherlands, July 23‐27, 2018.

[12] Pan S , Xu J , Han X , Zhang J , Hu Q , Chu Z , et al. Internet‐based sex‐seeking behavior promotes HIV infection risk: a 6‐year serial cross‐sectional survey to MSM in Shenyang, China. Biomed Res Int. 2016;2016:2860346. 28105415 10.1155/2016/2860346 PMC5220408

[13] Yang Z , Zhang S , Dong Z , Jin M , Han J . Prevalence of unprotected and intercourse in men who have sex with men recruited online versus offline: a meta – analysis. BMC Public Health. 2014;14:508. 24885058 10.1186/1471-2458-14-508 PMC4070357

[14] Baral S , Trapence G , Motimedi F , Umar E , Iipinge S , Dausabet F , et al. HIV prevalence, risks for HIV infection, and human rights among men who have sex with men (MSM) in Malawi, Namibia, and Botswana. PLoS ONE. 2009;4:e4997. 19325707 10.1371/journal.pone.0004997 PMC2657212

[15] Stahlman S , Nowak RG , Liu H , Crowell TA , Ketende S , Blattner WA , et al. Online sex‐seeking among men who have sex with men in Nigeria: implications for online intervention. AIDS Behav. 2017;21(11):3068–77. 27233248 10.1007/s10461-016-1437-3 PMC5124554

[16] National AIDS and STI Control Programme and University of Manitoba . Virtual mapping: harnessing online social networks to reach Men having sex with Men in Kenya; Evidence brief. Ministry of Health, Kenya. 2018 [cited 2019 Sep 13] Available from: http://www.childrenandaids.org/sites/default/files/2018‐11/Virtual%20mapping%20‐%20Harnessing%20online%20social%20networks%20to%20reach%20men%20who%20have%20sex%20with%20men%20in%20Kenya.pdf

[17] Geibel S , King'ola N , Temmerman M , Luchters S . The impact of peer outreach on HIV knowledge and prevention behaviours of male sex workers in Mombasa, Kenya. Sex Transmit Infect. 2012;88(5):357–62. 10.1136/sextrans-2011-050224 22332149

[18] Nzioka Denis , Mwanzia E .HIV services a click away! Online booking in Kenya. Taking HIV outreach and service delivery online – Results from Asia, Africa and the Caribbean, LINKAGES Webinar series. 18 September 2019 [cited 2020 Feb 24]. Available from: https://gallery.mailchimp.com/017a363ed67b2aa8d9ffcaa4a/files/e970f11b‐dad9‐4ccd‐b2c8‐3c5d13fcc5d9/Nzioka_and_Mwanzia_Kenya_Step1_Online_booking_v4.pdf

[19] Baggaley RF , White RG , Boily MC . HIV transmission risk through anal intercourse: systematic review, meta‐analysis and implications for HIV prevention. Int J Epidemiol. 2010;39(4):1048–63. 20406794 10.1093/ije/dyq057 PMC2929353

[20] Kunzweiler CP , Bailey RC , Okall DO , Graham SM , Mehta SD , Otieno FO . Factors associated with prevalent HIV infection among Kenyan MSM: The Anza Mapema study. J Acquir Immune Defic Syndr. 2017;76(3):241–9. 28746167 10.1097/QAI.0000000000001512

[21] McKinnon LR , Gakii G , Juno JA , Izulla P , Munyao J , Ireri N , et al. High HIV risk in a cohort of male sex workers from Nairobi, Kenya. Sex Transmit Infect. 2014;90(3):237–42. 10.1136/sextrans-2013-051310 24337729

[22] Geibel S , Luchters S , King'Ola N , Esu‐Williams E , Rinyiru A , Tun W . Factors associated with self‐reported unprotected anal sex among male sex workers in Mombasa, Kenya. Sex Transmit Dis. 2008;35(8):746–52. 10.1097/OLQ.0b013e318170589d 18650772

[23] Bhattacharjee P , Rego D , Musyoki H , Becker M , Pickles M , Isac S , et al. Evaluation of community‐based HIV self‐testing delivery strategies on reducing undiagnosed HIV infection, and improving linkage to prevention and treatment services, among men who have sex with men in Kenya: a programme science study protocol. BMC Public Health. 2019;19:986. 31337368 10.1186/s12889-019-7291-2 PMC6652006

[24] National AIDS and STI Control Programme (NASCOP), Kenya . Key Population mapping and size estimation in selected counties in Kenya: phase 1‐ key findings. Kenya: Ministry of Health; 2019 [cited 2019 Sep 9]. Available from: https://hivpreventioncoalition.unaids.org/wp-content/uploads/2020/02/KPSE-Phase1-Final-Report.pdf

[25] Paz‐Bailey G , Hoots BE , Xia M , Finlayson T , Prejean J , Purcell DW , et al. Trends in Internet Use Among Men Who Have Sex With Men in the United States. J Acquir Immune Defic Syndr. 2017;75 Suppl 3:S288–95. 28604430 10.1097/QAI.0000000000001404 PMC5871925

[26] Wheeler J , Anfinson K , Valvert D , Lungo S . Is violence associated with increased risk behavior among MSM? Evidence from a population‐based survey conducted across nine cities in Central America. Glob Health Action. 2014;7:24814. 25361722 10.3402/gha.v7.24814 PMC4212078

[27] Janulis P , Phillips G , Birkett M , Mustanski B . Sexual networks of racially diverse young MSM differ in racial homophily and not concurrency. J Acquir Immune Defic Syndr. 2018;77(5):459–66. 29280767 10.1097/QAI.0000000000001620 PMC5844824

[28] Smith AMA , Grierson J , Wain D , Pitts M , Pattison P . Associations between sexual behavior of men who have sex with men and the structure and composition of their sexual networks. Sex Transmit Infect. 2004;80:455–8. 10.1136/sti.2004.010355 PMC1744944 15572613

[29] Wohlfeiler D , Potterat JJ . Using gay men’s sexual networks to reduce sexually transmitted disease (STD)/human immunodeficiency virus (HIV) transmission. Sex Transmit Dis. 2005;32 10 Suppl:S48–52. 10.1097/01.olq.0000175394.81945.68 16205293

[30] Nöstlinger C , Rosińska M , Sherriff N , Gios L , Dias SF , Gama AF , et al. Behavioural and demographic correlates of undiagnosed HIV infection in a MSM sample recruited in 13 European cities. BMC Infect Dis. 2018;18:368. 30081839 10.1186/s12879-018-3249-8 PMC6080551

[31] Sandfort Theo G M , Knox Justin , Collier Kate L , Lane Tim . and Vasu Reddy. HIV testing practices of South African township MSM in the era of expanded access to ART. AIDS Behav. 2015;19(3):561–74. 25103866 10.1007/s10461-014-0843-7 PMC4320981

[32] Sandfort TGM , Dominguez K , Kayange N , Ogendo A , Panchia R , Chen YQ , et al. HIV testing and the HIV care continuum among sub‐Saharan African men who have sex with men and transgender women screened for participation in HPTN 075. PLoS ONE. 2019;14:e0217501. 31150447 10.1371/journal.pone.0217501 PMC6544251

[33] Lehmiller JJ , Ioerger M . Social networking smartphone applications and sexual health outcomes among men who have sex with men. PLoS ONE. 2014;23:e86603. 10.1371/journal.pone.0086603 PMC3900560 24466166

[34] Patel Rupa R , Curoe Katherine A , Chan Philip A . Undetectable equals untransmittable: a game changer for HIV prevention. Clin Chem. 2020;66(3):406–7. 32109297 10.1093/clinchem/hvz010

[35] Carballo–Dieguez A , Miner M , Dolezal C , Rosser BRS , Jacob S . Sexual negotiation, HIV status disclosure and sexual risk behavior among latino men who is the internet to seek sex with other men. Arch Sex Behav. 2006;35(4):473–81. 16933107 10.1007/s10508-006-9078-7

[36] Bowen AM , Williams ML , Daniel CM , Clayton S . Internet based HIV prevention research targeting rural MSM: feasibility, acceptability, and preliminary efficacy. J Behav Med. 2008;31(6):463–77. 18770021 10.1007/s10865-008-9171-6 PMC2614302

[37] OHTN Rapid Response Service . Rapid Review: HIV Prevention for Men who have Sex with Men. Toronto, ON: Ontario HIV Treatment Network; 2010.

[38] Rosenberger JG , Reece M , Novak DS , Mayer KH . The Internet as a valuable tool for promoting a new framework for sexual health among gay men and other men who have sex with men. AIDS Behav. 2011;15 Suppl 1:S88–90. 21331800 10.1007/s10461-011-9897-y

[39] Cao B , Gupta S , Wang J , Hightow‐Weidman LB , Muessig KE , Tang W , et al. Social media interventions to promote HIV testing, linkage, adherence, and retention: systematic review and meta‐analysis. J Med Int Res. 2017;19:e394. 10.2196/jmir.7997 PMC5722976 29175811

[40] Wu D , Tang W , Lu H , Zhang TP , Cao B , Ong JJ , et al. Leading by example: web‐based sexual health influencers among men who have sex with men have higher HIV and Syphilis testing rates in China. J Med Int Res. 2019;21:e10171. 10.2196/10171 PMC6360381 30664490

[41] Kubichek K , Carpineto J , McDavitt B , Weiss G , Kipke MD . Use and perceptions of the internet for sexual information and partners: a study of young men who have sex with men. Arch Sex Behav. 2011;40(4):803–16. 20809373 10.1007/s10508-010-9666-4 PMC3000442

[42] Nugroho A , Erasmus V , Zomer TP , Wu Q , Richardus JH . Behavioural interventions to reduce HIV risk behavior of MSM and transwomen in Southeast Asia: a systematic review. AIDS Care. 2017;29(1):98–104. 27329745 10.1080/09540121.2016.1200713

[43] Nyato D , Kuringe Evodius , Drake Mary , Casalini Caterina , Nnko Soori , Shao Amani , et al. Participants’ accrual and delivery of HIV prevention interventions among men who have sex with men in sub‐Saharan Africa: a systematic review. BMC Public Health. 2018;18:370. 29554867 10.1186/s12889-018-5303-2 PMC5859521

[44] Wei C , Lim SH , Guadamuz TE , Koe S . Virtual vs. physical spaces: which facilitates greater HIV risk taking among men who have sex with men in East and South‐East Asia? AIDS Behav. 2014;18(8):1428–35. 24077974 10.1007/s10461-013-0628-4 PMC4053516

[45] Lee S‐S , Lam ANS , Lee C‐K , Wong N‐S . Virtual versus physical channel for sex networking in men having sex with men of sauna customers in the City of Hong Kong. PLoS ONE. 2012;7:e31072. 22348038 10.1371/journal.pone.0031072 PMC3277598

[46] Behanzin L , Diabate S , Minani I , Lowndes CM , Boily MC , Labb AC , et al. Assessment of HIV related risky behaviour: a comparative study of face‐to‐face interviews and polling booth surveys in the general population of Cotonou, Benin. Sex Transmit Infect. 2013;89:595‐601. 10.1136/sextrans-2012-050884 PMC3800174 23723251

[47] Smith AD , Muhaari A , Agwanda C , Kowuor D , Van der Elst E , Davies A , et al. Heterosexual behaviours among men who sell sex to men in Coastal Kenya. AIDS. 2015;29(0 3):S201–10. 26565965 10.1097/QAD.0000000000000889 PMC4706370

