# Supplementary material for: HIV prevalence, testing and treatment among men who have sex with men through engagement in virtual sexual networks in Kenya: a cross‐sectional bio‐behavioural study
Source: J Int AIDS Soc. 2020 Jun 26;23(Suppl 2):e25516. doi: 10.1002/jia2.25516 (PMC7319161; doi:10.1002/jia2.25516)
Supplement: Supplementary file 1 — Appendix S1. Survey questionnaire for baseline and end line survey. [file JIA2-23-e25516-s001.pdf]

Annexure 3: Survey questionnaire for baseline and end line survey

FORM NO

Designing a community-based programmatic research to evaluate HIV Self-Testing among MSM in Kenya

DATE:

SITE NAME AND CODE:____

TYPE OF SITE: PHYSICAL VIRTUAL

TYPOLOGY*:______________________________

TYPE OF SELECTION: PRIMARY SECONDARY

COUNTY: ______________________________ SUB

COUNTY :______________________________

INTERVIEWER NAME : ______________________________ SIGNATURE:

____

SUPERVISOR NAME:______________________________

SIGNATURE:______________________________

1. Street; 2. Home; 3. Bus/taxi/truck stand; 4. Bar/restaurant; 5. Lodge/hotel; 6. Massage parlor; 7. Markets. 8. Through middleman; 9. Social gatherings; 10. Phone/mobile; 11. Internet; 12. Facebook; 13. Whatsapp; 14. Other (specify)…………………

Sl	QUESTION	CATEOGRY	SKIP
1	How old are you?	AGE IN COMPLETED YEARS	
2	What is the highest level of education you attended?	NO FORMAL EDUCATION .... 1<br>PRIMARY .... 2<br>SECONDARY .... 3<br>TERTIARY/COLLEGE/UNIVERSITY................................ 4<br>OTHERS(SPECIFY) 5	
3	What is your marital status?	SINGLE... 1<br>MARRIED... 2<br>DIVORCED/WIDOWED/SEPARATED .... 3<br>NO ANSWER...99	
4	Who are you currently living with?<br>**MORE THAN ONE OPTION POSSIBLE**	LIVING ALONE.. A<br>PARENTS... B<br>BROTHER(S) AND/OR SISTER....................................... C<br>OTHER FAMILY.. D<br>MALE SEXUAL PARTNE..E<br>FEMALE SEXUAL PARTNER..F<br>CHILDREN.. G<br>OTHERS(SPECIFY) .... X<br>NO ANSWER ....Z	
5	What is your main occupation/employment?	BUSINESS .... 1<br>CONSTRUCTION WORKER .... 2<br>FACTORY WORKER .... 3<br>FARMING/AGRICULTURAL WORKER .... 4<br>FISHERMAN/SEAFARER .... 5<br>GOVERNMENT WORKER .... 6<br>HAIRDRESSER/BEAUTICIAN/MASSEUSE .... 7<br>SHOP WORKER... 8<br>STREET VENDOR/CASUAL LABOURER .... 9<br>TOURISM/TRAVEL AGENT/TOUR GUIDE ....10<br>WAITER-WAITRESS/BARTENDER/<br>HOTEL EMPLOYEE 11	

		SEX WORK .... 12<br>NO INCOME .... 13 →<br><br>OTHER (SPECIFY): .... 97<br>DON'T KNOW .... 98<br>NO ANSWER` 99	7
6	What is your average monthly income?	SHILLING ☐☐☐☐☐☐	
7	In which County / Sub county do you live?	COUNTY:________ ☐☐<br><br>SUBCOUNTY:________ ☐☐	
8	How do you predominantly describe your sexual orientation/identity?	GAY .... 1<br>BI SEXUAL .... 2<br>MSM/MSW .... 3<br>OTHERS .... 7<br>NO ANSWER 99	
9	How do you describe your sexual preference/orientation/identity?	PREDOMINANTLY RECEPTIVE (BOTTOM/HANITHI) .... 1<br>PREDOMINANTLY INSERTIVE (TOP/BASHA) .... 2<br>BOTH RECEPTIVE AND INSERTIVE (VERSATILE) .... 3<br>BISEXUAL .... 4<br>TRANSGENDER .... 5<br><br>OTHERS (SPECIFY) .... 97<br>NO ANSWER 99	
10	Who is/are the different person/s with whom you have disclosed your sexual preference/orientation/identity?<br><br>**MORE THAN ONE OPTION POSSIBLE**	SPOUSE .... A<br>FAMILY MEMBERS .... B<br>FRIENDS .... C<br>NEIGHBOURS .... D<br>OTHER MSMS .... E<br>HEALTH CARE PROVIDERS .... F<br>OTHERS (SPECIFY)________X<br><br>NONE .... Y<br>NO ANSWER .... Z	
11	How old were you when you had your first sex (anal, oral or vaginal)?	AGE ☐☐<br><br>NEVER HAD SEX .... 95 →<br>DON'T KNOW .... 98<br>NO ANSWER 99	END
12	How old were you when you had you had first sex (anal or oral) with a man?	AGE ☐☐<br><br>NEVER HAD ANAL .... 95 →<br>DON'T KNOW .... 98<br>NO ANSWER 99	END
13	Have you ever sex (anal or oral) with a man in the past 12 months?	YES .... 1<br>NO .... 0 }<br>NO ANSWER 99	END
14	How many different male sexual partners have you had in the last one month?	NUMBER OF PARTNERS ☐☐<br><br>NONE .... 00 →<br>DON'T KNOW .... 98<br>NO ANSWER 99	END
15	How many different male sexual partners have you had in the last one week?	NUMBER OF PARTNERS ☐☐<br><br>NONE .... 00<br>DON'T KNOW .... 98<br>NO ANSWER 99	
16	Do you receive money/gifts in exchange of sex with another man?	YES .... 1<br>NO .... 0<br>NO ANSWER 99	
17	Which are the different places/locations through which	STREET .... A<br>HOME .... B<br>BUS/TAXI/TRUCK STAND .... C<br>BAR/RESTAURANT .... E<br>LODGE/HOTEL .... F	

	you have met other male sexual partners?<br>MORE THAN ONE OPTION POSSIBLE	MASSAGE PARLOR................... .... G<br>MARKETS.. H<br>THROUGH MIDDLEMAN............ .... I<br>SOCIAL GATHERINGS................ .... J<br>PHONE/MOBILE..................... .... K<br>INTERNET/WEBAPP..L<br>FACEBOOK .... M<br>WHATSAPP.. N<br><br>OTHER (SPECIFY)..................... .... X<br>NO ANSWER................................. ....Z	
18	Among the above places, which is the most common place from where you get your most male sexual partners?	CODE THE CORRESPONDING FROM ABOVE .... ☐	

19	Now I would like to ask you few questions about the 3 most recent male sexual partners you had;			
S.N		MOST RECENT PARTNER	SECOND MOST RECENT PARTNER	THIRD MOST RECENT PARTNER
A	Where did you meet him for the first time?<br>**USE CODES FROM QUESTION 17**	☐	☐	☐
B	When you first had sex with this partner, how old were you?	AGE ☐☐	AGE ☐☐	AGE ☐☐
C	For how long have you had/ having sexual relations with this partner?	MONTHS....1<br>YEARS ....2<br>DON'T KNOW ....<br>98<br>NO ANSWER ....<br>99	MONTHS....1<br>YEARS ....2<br>DON'T KNOW ....<br>98<br>NO ANSWER99	MONTHS....1<br>YEARS ....2<br>DON'T KNOW ....<br>98<br>NO ANSWER99
D	In the past 30 days, how many times have you had sex with this partner?	NO. OF TIMES	NO. OF TIMES	NO. OF TIMES ☐☐
E	The last time when you had sex with this person, were you top or bottom?	TOP ....<br>1<br>BOTTOM ....<br>2<br>NO ANSWER ....<br>99	TOP ....<br>1<br>BOTTOM ....<br>2<br>NO ANSWER 99	TOP ....<br>1<br>BOTTOM ....<br>2<br>NO ANSWER 99
F	The last time when you had sex with this partner, was a condom used?	YES ....<br>1<br>NO ....<br>0<br>DON'T KNOW ....<br>98<br>NO ANSWER ....<br>99	YES....................... 1<br>NO .... 0<br>DON'T KNOW ...... 98<br>NO ANSWER 99	YES....................... 1<br>NO .... 0<br>DON'T KNOW ...... 98<br>NO ANSWER 99
G	What motivated you to have sex with this partner?	MONEY ....<br>1<br>PLEASSURE ....<br>2<br>OTHERS.........................<br>3	MONEY ....<br>1<br>PLEASSURE ....<br>2<br>OTHERS.........................<br>3	MONEY ....<br>1<br>PLEASSURE ....<br>2<br>OTHERS.........................<br>3

		NO ANSWER 9	NO ANSWER 9	NO ANSWER 9
H	The last time when you had sex with this partner, did you pay/receive money/gifts?	YES, MONEY .... 1<br>YES, GIFT .... 2<br>NO .... 3<br>NO ANSWER 9	YES, MONEY .... 1<br>YES, GIFT .... 2<br>NO .... 3<br>NO ANSWER 9	YES, MONEY .... 1<br>YES, GIFT .... 2<br>NO .... 3<br>NO ANSWER 9

HIV TESTING

SI	QUESTION	CATEGRY	SKIP
20	Have you ever been tested for HIV?	YES.......................................1<br>NO ....0<br>DON'T KNOW...............................98<br>NO ANSWER 99	→ 32
21	How many years/months ago was the first time you tested for HIV?	YEARS AGO [ ][ ]<br>MONTHS AGO [ ][ ]<br>DON'T KNOW...............................98<br>NO ANSWER 99	
22	When did you have your MOST recent HIV test?	NUMBER OF MONTHS AGO [ ][ ]<br>DON'T KNOW...............................98<br>NO ANSWER 99	
23	How many times have you tested for HIV in the last 12 months?	NUMBER OF TIMES [ ][ ]<br>NONE.......................................00<br>DON'T KNOW...............................98<br>NO ANSWER 99	
24	Which was the place where you visited for the most recent HIV test?	GOVERNMENT FACILITY.......................1<br>PRIVATE FACILITY...........................2<br>MSM FRIENDLY CLINICS/DICE.................3<br>SELF TEST.................................4<br>OTHERS (SPECIFY)..........................5<br>NO ANSWER 99	
25	What was the test result of your most recent HIV test?	HIV-POSITIVE ....1<br>HIV-NEGATIVE ....2<br>INDETERMINATE..............................3<br>DID NOT RECEIVE RESULT ....4<br>DON'T KNOW.................................5<br>REFUSE TO ANSWER ....5<br>DON'T KNOW................................ 98	
26	Are you enrolled in any care and treatment center (CCC)?	YES.......................................1<br>NO ....0<br>NO ANSWER 99	35 →
27	How long have you been enrolled in any care and treatment center (CCC)?	NUMBER OF MONTHS AGO [ ][ ]	
28	Have you ever been on ART?	YES.......................................1<br>NO ....0<br>NO ANSWER 99	35 →
29	How long have you been on ART?	NUMBER OF MONTHS [ ][ ]	

30	Are you currently on ART?	YES....1<br>NO....0<br>NO ANSWER....99	35 → (NO)
31	Have you ever missed taking ARV in the past one month?	YES....1<br>NO....0<br>NO ANSWER....99	35 → (NO)
32	What was the reason/s for not undergoing HIV testing?<br><br>**MORE THAN ONE OPTION POSSIBLE**	I DON'T THINK I HAVE HIV/NO REASON TO TEST....A<br>AFRAID OF LEARNING HIV STATUS....B<br>LACK OF CONFIDENTIALITY....C<br>TAKE TOO MUCH TIME....D<br>NOT EASILY ACCESSIBLE....E<br>NOT AWARE OF A PLACE....F<br>DID NOT WANT TO GO TO A FACILITY THEN....G<br>OTHERS (SPECIFY)....X<br>NO ANSWER....Z	
33	Do you know a place where HIV testing can be done?	YES....1<br>NO....0<br>DON'T KNOW....98<br>NO ANSWER....99	35 →
34	Which is/are the place/s where you can visit for HIV testing?<br><br>**MORE THAN ONE OPTION POSSIBLE**	GOVERNMENT FACILITY....A<br>PRIVATE FACILITY....B<br>MSM FRIENDLY CLINICS....C<br>SELF TESTS....D<br>OTHERS (SPECIFY)....X<br>NO ANSWER....Z	

SI	QUESTION	CATEGRY	SKIP
35	Have you ever heard of HIV self-testing?	YES....1<br>NO....0<br>DON'T KNOW....98<br>NO ANSWER....99	42 →
36	Have you ever done HIV Self-Testing ?	YES....1<br>NO....0<br>DON'T KNOW....8<br>NO ANSWER....9	41 →
37	The last time when you used HIV self-testing, how long ago was it?	MONTHS AGO [ ][ ]	
38	The last time when you used HIV self- testing, where did you test?	HOME....1<br>FRIENDS PLACE....2<br>MSM FRIENDLY CLINIC....3<br>GOVERNMENT FACILITY....4<br>PRIVATE FACILITY....5<br>OTHERS (SPECIFY)....7<br>NO ANSWER....9	
39	The last time when you used HIV self-testing, who assisted you for testing?	PARTNER....1<br>PEER EDUCATOR/ OUTREACH WORKER....2<br>COUNSELOR....3<br>HEALTH CARE PROVIDER....4<br>OTHERS (SPECIFY)....7<br>NO ONE----98<br>NO ANSWER....99	
40	The last time when you used HIV self- testing, where did you receive the test kit?	PHARMACY....1<br>FRIENDS....2<br>MSM FRIENDLY CLINIC....3<br>GOVERNMENT FACILITY....4<br>PRIVATE FACILITY....5<br>OTHERS (SPECIFY)....7<br>NO ANSWER....9	
41	According to you what are the advantages of using HIV self-testing?<br><br>**MORE THAN ONE RESPONSE**	PRIVACY....A<br>CONVENIENCE....B<br>NOONE KNOW THE STATUS....C<br>NO BLOOD SAMPLE/PRICK REQUIRED....D<br>NO NEED TO VISIT HEALTH FACILITY....E<br>CAN DO AT HOME/AT CONVENIENCE....F<br>OTHERS (SPECIFY)....X<br>NO ADVANTAGE....Y	

		NO ANSWER ....Z	
**INSTRUCTION: IF Q35 IS NO, (NOT HEARD OF SEL-TESING), INTERVIEWER TO EXPLAIN THE PARTICIPANT ABOUT THE HIV SELF-TESTING**			
42	The next time when you go for HIV testing, which method/place would you go?<br><br>**THE INTERVIEWER ALSO EXPLAINS THE HIV SELF TESTING**	HIV SELF TESTING ....1<br>MSM FRIENDLY CLINIC...2<br>GOVERNMENT FACILITY...3<br>PRIVATE FACILITY..4<br>OTHERS (SPECIFY)..7<br>NO ANSWER 9	
43	How often would you like to test for HIV using HIV self-testing?	ATLEAST ONCE IN 3 MONTHS....................................1<br>ATLEAST ONCE IN 6 MONTHS....................................2<br>ATLEAST ONCE IN AN YEAR..3<br>LESS THAN ONCE A YEAR ....4<br>DON'T KNOW...98<br>NO ANSWER 99	
44	What do you think are the main disadvantages of HIV self-testing?<br><br>**MORE THAN ONE OPTION POSSIBLE**	NO DISADVANTAGE ....A<br>LACK OF POST TEST CONSELING INCREASE<br>DISTRESS ....B<br>POSSIBILITY OF SELF-HARM ....C<br>POSSIBILITY OF HARMING OTHERS........................... D<br>REDUCED CHANCE OF DISCLOSURE/<br>ENROLLMENT IN CARE ....E<br>OTHERS (SPECIFY)..X	
45	How would you like to receive HIV self-test kits?<br><br>**MORE THAN ONE OPTION POSSIBLE**	PEER EDUCTOR/ORW ....A<br>HTS ....B<br>NGO/PROGRAMS...C<br>PHARMACY .... D<br>FRIENDS..E<br>OTHERS (SPECIFY)..X<br>NO ANWER ....Z	

PROGRAM EXPOSURE

46	Have you ever heard of an organization/NGO in this County offering health services for men who have sex with men?	YES...1<br>NO ....0<br>DON'T KNOW...98<br>NO ANSWER 99	
47	Have you ever been contacted by a peer educator/outreach worker in the last 3 months?	YES...1<br>NO ....0<br>DON'T KNOW...98<br>NO ANSWER 99	→ 50
48	The last time when you were contacted by a peer educator/outreach worker, how/where did he contact you?	AT HOME ....1<br>AT PHYSICAL LOCATION OF MSM ....2<br>THROUGH PHONE..3<br>OTHER...97<br>NO ANSWER 99	
49	The last time when you were contacted by a peer educator/outreach worker, what services did have you received?	CONDOM ....A<br>LUBRICANTS...B<br>HIV/STI EDUCATION...C<br>HIV TESTING .... D<br>OTHERS (SPECIFY)..X<br>NO ANSWER ....Z	
50	Which source/s would you like to receive HIV related information/services?	PEER EDUCATOR/ORW ....A<br>INTERNET/WEBSITE ....B<br>PHONE ....C<br>ELECTRONIC/PRINT MEDIA....................................... D<br>PEERS/FRIENDS...E<br>OTHERS (SPECIFY)..X<br>NO ANSWER ....Z	
51	Have you ever visited a clinic or drop-in Centre in or around your town/ub county/ county that provides health information or services to MSM/MSW in the past 3 months?	YES...1<br>NO ....0<br>DON'T KNOW...98<br>NO ANSWER 99	→ 53

52	The last time when you visited a clinic/drop-in-center, what service/s did you received?	CONDOM ....A<br>LUBRICANTS....B<br>STI SCREENING....C<br>STI TREATMENT .... D<br>HIV TESTING .... E<br>HIV SELF-TESTING KIT .... F<br>COUNSELING .... G<br>ART----H<br>OTHERS (specify) ....X<br>NO ANSWER ....Z	
53	Have you ever registered with a program/NGO that provides exclusive services to MSM community?	YES....1<br>NO ....0<br>DON'T KNOW....98<br>NO ANSWER 99	YES → 55
54	Would you like to be registered/ engaged with the programme?	YES....1<br>NO ....0<br>DON'T KNOW....98<br>NO ANSWER 99	

ALCOHOL, DRUG AND VIOLENCE AND STIGMA

55	During the past month, how often did you consumed drinks containing alcohol?	NEVER....1<br>EVERY DAY....2<br>AT LEAST ONCE A WEEK....3<br>LESS THAN ONCE A WEEK....4<br>NOT IN THE PAST MONTH....5<br><br>DON'T KNOW....98<br>NO ANSWER....99	
56	How often were you or your clients/partner under the influence of alcohol while having sex?	EVERYTIME....1<br>OFTEN....2<br>SOMETIME....3<br><br>NEVER....97<br>NO ANSWER....99	
57	Some people consume drugs for no-medical reasons, like marijuana, heroin, amphetamine, etc to feel good, get high, fly, trip or fantasies. Have you ever consumed drugs like these, even one time?	YES....1<br>NO....0<br><br>DON'T KNOW....98<br>NO ANSWER....99	
58	Have you ever injected drugs for non-medical reasons?	YES....1<br>NO....0<br><br>DON'T KNOW....98<br>NO ANSWER....99	NO → 61
59	In the past 12 months, have you ever injected drugs for non-medical reasons?	YES....1<br>NO....0<br><br>DON'T KNOW....98<br>NO ANSWER....99	
60	When you injected drugs in the past 12 months, did you share needle with any one?	YES ....1<br>NO ....0<br>DON'T KNOW....98<br>NO ANSWER 99	
61	In the past 12 months, have you ever been verbally or physically assaulted/abused by family/ community?	YES....1<br>NO ....0<br>DON'T KNOW....98<br>NO ANSWER 99	NO/DON'T KNOW → 63
62	The last time you experienced a verbal or physical assault/abuse, who perpetrated this?	PARTNER....1<br>FAMILY MEMBERS ....2<br>FRIENDS....3<br>COLLEAGUES....4<br>NEIGHBOURS ....5<br>POLICE/LAW ENFORCEMENT....6<br>OTHERS (specify) ....7<br>NO ANSWER 9	

63	In the past 12 months, have you ever been sexually assaulted/abused by someone?	YES....1<br>NO....0<br>DON'T KNOW....98<br>NO ANSWER....99	65 →
64	The last time you experienced a sexual assault/abuse, who perpetrated this?	PARTNER....1<br>FAMILY MEMBERS....2<br>FRIENDS....3<br>COLLEAGUES....4<br>NEIGHBOURS....5<br>POLICE/LAW ENFORCEMENT....6<br>OTHERS (SPECIFY)....7<br>NO ANSWER....9	
65	In the past 12 months, have you ever been excluded from social gatherings/family/ friends/community?	YES....1<br>NO....0<br>DON'T KNOW....98<br>NO ANSWER....99	67 →
66	The last time when you experienced an exclusion from social gatherings/family/friends/ community, who perpetrated this?	FAMILY MEMBERS....1<br>FRIENDS....2<br>COLLEAGUES....3<br>NEIGHBOURS....4<br>POLICE/LAW ENFORCEMENT....5<br>OTHERS (SPECIFY)....7<br>NO ANSWER....9	
67	In the past 12 months, have you ever been rejected by health care provider because of HIV status?	YES....1<br>NO....0<br>DON'T KNOW....98<br>NO ANSWER....99	69 →
68	The last time when you were rejected by a health care provider, what type of facility was it?	GOVERNMENT FACILITY....1<br>PRIVATE FACILITY....2<br>NGO RUN FACILITY....3<br>OTHERS (SPECIFY)....7<br>NO ANSWER....9	
90	In the past 12 months, have you ever been harassed/arrested by a law enforcement agencies?	YES....1<br>NO....0<br>DON'T KNOW....98<br>NO ANSWER....99	

THANKYOU

Kiambatisho 2: Dodoso la utafiti wa msingi na wa mwisho.

FOMU NA | | | |

Kuunda Utafiti wa kijamii ili kutathmini kujipima VVU miongoni mwa Wanaume wanaofanya ngono na Wanaume wenging nchini Kenya

SITE NAME AND CODE:____ DATE:

TYPE OF SITE: PHYSICAL VIRTUAL

TYPOLOGY:

COUNTY: ____ SU

COUNTY :____

INTERVIEWER NAME : ____ SIGNATURE:

____

SUPERVISOR NAME:____

SIGNATURE:____

1. Street; 2. Home; 3. Bus/taxi/truck stand; 4. Bar/restaurant; 5. Lodge/hotel; 6. Massage parlor; 7. Markets. 8. Through middleman; 9. Social gatherings; 10.
Phone/mobile; 11. Internet; 12. Facebook; 13. Whatsapp; 14. Other (specify)…………………

SI	SWALI	CATEOGRY	RUKA
1	Una miaka mingapi?	Umri(Miaka)	
2	Kiwango cha juu kabisa cha masomo ulichokamilisha ni kipi?	SINA ELIMU MAALUM .... 1<br>SHULE YA MSINGI.. 2<br>SHULE YA UPILI.. 3<br>SHULE ZA TAALUMA (TECHNICAL SCHOOL, CHUO KIKUU 4<br>NYINGINEYO, ELEZEA 5	
3	Hali yako ya ndoa ni ipi?	SIJAWAHI OA/OLEWA .... 1<br>NIMEOA/OLEWA .... 2<br>NIMETALIKI/NIMEFIWA/TUMEACHANA.................... 3<br>SINA JIBU/SITAKI KUJIBU 99	
4	Hivi sasa unaishi na nani?<br>**MAJIBU MENGI YANAKUBALIKA**	NAISHI PEKEE YANGU..A<br>WAZAZI...B<br>NDUGU NA/AU DADA...C<br>WANAFAMILIA WENGINE..D<br>MPENZI WA KIUME...E<br>MPENZI WA KIKE...F<br>MTOTO...G<br>WENGINEO (ELEZA) ....X<br>SINA JIBU..Z	
5	Je unafanya kazi gani maalum?	BIASHARA.. 1<br>MFANYIKAZI WA UJENZI.. 2<br>MFANYIKAZI WA KIWANDANI..3<br>MKULIMA/MFANYIKAZI WA HUDUMA ZA KILIMO.4<br>MVUVI. ....5<br>MFANYIKAZI WA SERIKALI..6<br>MSUSI...7<br>MFANYIKAZI WA DUKA.........................8<br>Mchuuzi..9<br>Mhudumu wa watalii...................................10<br>Mhudumu wa hoteli.......................................11<br>Muuza ngono..12<br>NYINGINE (ELEZA.......................................)97<br>SIJUI...98<br>SINA JIBU...99	7
6	Je, Kwa takriban, mapato yako ya mwezi ni yapi?	SHILLINGI	

7	Unaishi kwenye Kaunti/Kaunti ndogo(subcounty) gani?	____________ KAUNTI<br>KAUNTI NDOGO:____________	
8	Mara Nyingi huwa unajitambuisha vipi kirasmi?	SHOGA .... 1<br>BI SEXUAL.......... 2<br>MSM/MSW .... 3<br>Vinginevyo.......... 7<br>SITAKI KUJIBU.......... 99	
9	How do you predominantly describe your sexual orientation/identity?	HANITHI.......... 1<br>BASHA .... 2<br>VERSATI .... 3<br>BISEXUAL .... 4<br>TRANSGENDER .... 5<br>NYINGINE (ELEZEA).......... 97<br>SINA JIBU.......... 99	
10	Umekuwa ukifanya ngono ya mkundu na wanaume kwa miaka ngapi?	IDADI YA MIAKA:<br>SIJAWAHI FANYA NGONO YA MKUNDU ....95 →<br>SIJUI.......... 98<br>SINA JIBU.......... 99	END<br>MWISHO
11	Who is/are the different person/s with whom you have disclosed your sexual preference/orientation/identity?<br>**MORE THAN ONE OPTION POSSIBLE**	MKE/MUME.......... A<br>JAMII YANGU.......... B<br>MARAFIKI.......... C<br>MAJIRANI.......... D<br>MSM WENZANGU.......... E<br>WAHUDUMU WA AFYA.......... F<br>NYINGINE (ELEZEA)____________X<br>HAKUNA .... Y<br>SITAKI KUJIBU.......... .Z	
12	Ulikuwa na miaka ngapi ulipofanya ngono kwa mara ya kwanza?	MIAKA:<br>SIJAWAHI FANYA NGONO ....95 →<br>SIJUI.......... ....98<br>SITAKI KUJIBU..........99	MWISHO
13	Ulikuwa na miaka ngapi ulipofanya ngono ya nyuma kwa mara ya kwanza na mwanaume?	MIAKA:<br>SIJAWAHI FANYA NGONO YA NYUMA..........95 →<br>SIJUI.......... ....98<br>SITAKI KUJIBU..........99	MWISHO
14	Je, umefanya ngono na mwanaume kwa mwaka moja uliopita?	NDIO ....1<br>LA..........0 }<br>SITAKI KUJIBU 99	MWISHO
15	Kwa mwezi moja uliopita, umekuwa na wapenzi wanaume wangapi?	IDADI YA WAPENZI:<br>HAKUNA .... 00 →<br>SIJUI.......... 98<br>SINA JIBU.......... 99	MWISHO
16	Huwa unalipisha ili ufanye ngono?	NDIO.......... 1<br>LA .... 0<br>SINA JIBU.......... 99	
17	Kwa mwezi moja uliopita, je ni mahali/maeneo yapi amabayo	MTAA...........<br>NYUMBA	

	mlipatana na wapenzi wa kiume?	Eneo BASI/TEKSI/ama Truck zinabebea abiria/zinaegezwa.<br>BA/Hoteli<br>Hoteli iliyo na malazi<br>MASSAGE PARLOR……………….<br>Sokoni<br>Kupitia mtu mwengine<br>Hadhara za jamii<br>Kupitia kwa simu<br>Kwenye Mtandao<br>Facebook<br>WHATSAPPPenginepo; elezea wapi<br>Sitaki kujibu	
