# Supplementary material for: HIV prevalence, testing and treatment among men who have sex with men through engagement in virtual sexual networks in Kenya: a cross‐sectional bio‐behavioural study
Source: J Int AIDS Soc. 2020 Jun 26;23(Suppl 2):e25516. doi: 10.1002/jia2.25516 (PMC7319161; doi:10.1002/jia2.25516)
Supplement: Supplementary file 2 — Table S1. Consumption of drugs (oral or injecting) and place of most recent test among MSM who meet sex partners solely via virtual sites versus solely via physical sites versus via both physical and virtual sites in Kenya, May to July 2019 [file JIA2-23-e25516-s002.docx]

|  | **Total (N=1195)** | **PMSM^a^ (N=176)** | **DMSM^a^ (N=765)** | **VMSM^a^ (N=254)** | **P Value** |
| --- | --- | --- | --- | --- | --- |
| **Ever consumed drugs to feel good, get high, fly, trip or fantasies, even one time** | 48.10% | 43.20% | 51.80% | 40.60% | 0.003 |
|  | **Total (N=1198)** | **PMSM (N=177)** | **DMSM (N=767)** | **VMSM (N=254)** | **P Value** |
| **Ever injected drugs for non-medical reasons )** | 2.80% | 2.30% | 3.40% | 1.60% | 0.284 |
|  | **Total (N=1195)** | **PMSM (N=175)** | **DMSM (N=766)** | **VMSM (N=254)** | **P Value** |
| **Place of most recent HIV test** |  |  |  |  |  |
| Government facility | 40.6% | 42.3% | 41.6% | 36.2% | 0.000 |
| Private facility | 14.0% | 16.0% | 12.7% | 16.5% |  |
| MSM friendly/DICE | 30.5% | 20.0% | 29.6% | 40.2% |  |
| HIV Self test | 2.1% | 1.7% | 2.2% | 2.0% |  |
| Others | 9.9% | 15.4% | 11.2% | 2.0% |  |
| Never tested | 3.0% | 4.6% | 2.6% | 3.1% |  |

**Appendix 2**

**Table S1: Consumption of drugs (oral or injecting) and place of most recent test among MSM who meet sex partners solely via virtual sites versus solely via physical sites versus via both physical and virtual sites in Kenya, May-July 2019**

MSM (men who have sex with men)

^a^ Participants who self-reported only virtual sites (internet/web app, Facebook, WhatsApp, mobile) as locations through which they met other male sex partners were defined as MSM who met sex partners solely using virtual sites (VMSM). Participants who reported using only physical sites (street, home, bus/ taxi stand/ lodge/ markets/ social gatherings) as locations through which they met other male sexual partners were defined as MSM who met sex partners solely using physical sites (PVSM). Participants who reported using both physical sites (street, home, bus/ taxi stand/ lodge/ markets/ social gatherings) and virtual sites (internet/web app, Facebook, WhatsApp, mobile) as locations through which they met male sexual partners were defined as MSM who met sex partners using dual sites (DMSM).
